# High Voltage Cycling Stability of LiF-Coated NMC811 Electrode

**DOI:** 10.1021/acsami.3c14394

**Published:** 2024-01-03

**Authors:** Princess Stephanie Llanos, Zahra Ahaliabadeh, Ville Miikkulainen, Jouko Lahtinen, Lide Yao, Hua Jiang, Timo Kankaanpää, Tanja M. Kallio

**Affiliations:** † Department of Chemistry and Materials Science, School of Chemical Engineering, Aalto University, 02150 Espoo, Finland; ‡ Department of Applied Physics, School of Science, Aalto University, 02150 Espoo, Finland; § OtaNano-Nanomicroscopy Center, Aalto University, 02150 Espoo, Finland; ∥ Umicore Finland Oy, 67101 Kokkola, Finland

**Keywords:** NMC811, high voltage cycling, electrode coating, LiF, atomic layer deposition, cathode electrolyte interface

## Abstract

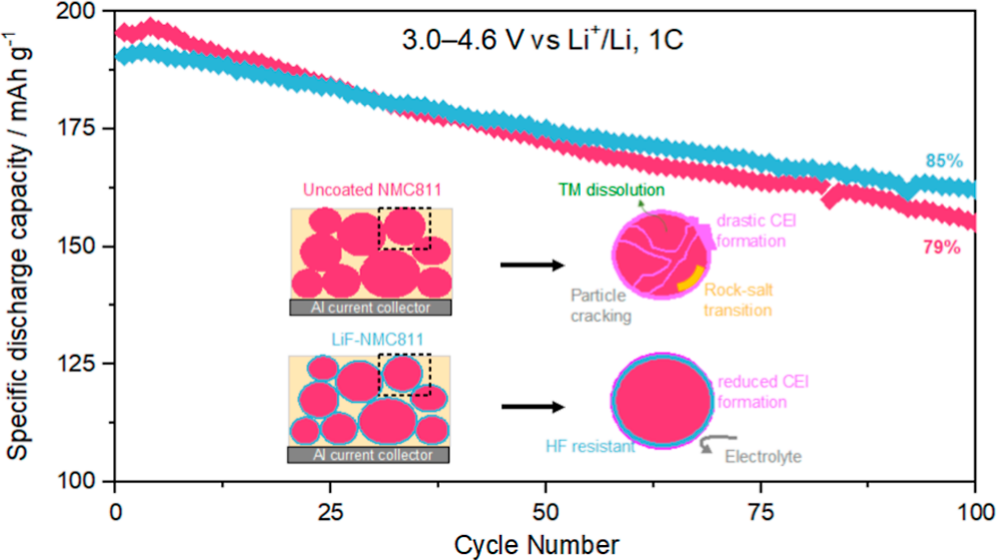

The development of LiNi_0.8_Mn_0.1_Co_0.1_O_2_ (NMC811) as a cathode material for high-energy-density lithium–ion batteries (LIBs) intends to address the driving limitations of electric vehicles. However, the commercialization of this technology has been hindered by poor cycling stability at high cutoff voltages. The potential instability and drastic capacity fade stem from irreversible parasitic side reactions at the electrode–electrolyte interface. To address these issues, a stable nanoscale lithium fluoride (LiF) coating is deposited on the NMC811 electrode via atomic layer deposition. The nanoscale LiF coating diminishes the direct contact between NMC811 and the electrolyte, suppressing the detrimental parasitic reactions. LiF-NMC811 delivers cycling stability superior to uncoated NMC811 with high cutoff voltage for half-cell (3.0–4.6 V vs Li/Li^+^) and full-cell (2.8–4.5 V vs graphite) configurations. The structural, morphological, and chemical analyses of the electrodes after cycling show that capacity decline fundamentally arises from the electrode–electrolyte interface growth, irreversible phase transformation, transition metal dissolution and crossover, and particle cracking. Overall, this work demonstrates that LiF is an effective electrode coating for high-voltage cycling without compromising rate performance, even at high discharge rates. The findings of this work highlight the need to stabilize the electrode–electrolyte interface to fully utilize the high-capacity performance of NMC811.

## 1 Introduction

The pursuit of zero transport emissions has been the stimulus for numerous studies on next-generation lithium–ion battery (LIB) materials.^1^ The transition to electromobility demands LIB technology shift to higher energy densities as electric vehicles (EVs) strive to drive longer distances with a single charge. Energy density improvement at the material and electrode levels allows a reduction of the number of cells needed to supply the same energy, thus reducing the overall cost.^2^ Layered lithium nickel manganese cobalt oxide (LiNi_*x*_Co_*y*_Mn_1–*x*–*y*_O_2_) (NMC) with high Ni content (*x* ≥ 0.6) is gaining attention as a cathode material for EV application due to its higher capacity compared with commercial NMC111 and NMC532 employed in current EV models.^3−5^ The higher capacity is due to the high Ni content, which brings more redox reactions of Ni^2+^/Ni^3+^/Ni^4+^, the major contributors to NMC capacity.^2,6,7^ NMC811 is especially attractive as it can deliver capacities exceeding 200 mA h g^–1^ at a wide electrochemical window.^8^

A higher cutoff voltage allows for the maximum utilization of available capacity. However, delithiation at potentials above 4.3 V vs Li/Li^+^ for NMC811 can result in several material degradation mechanisms, including electrolyte decomposition, cathode–electrolyte interphase (CEI) growth, transition metal (TM) dissolution, irreversible phase transition, and particle cracking.^2,3,6,8−14^ These issues are related and mutually result in active material loss, inhibited Li^+^ transport, and increased cell impedance, all of which contribute to poor capacity retention during prolonged cycling, inferior rate capability, and thermal instability.^2,14^ The limited operating voltage condition hampers the large-scale commercialization of NMC811-based LIB, as higher energy density should be coupled with a longer cycling lifetime when it comes to EV applications.^5^

The aforementioned degradation mechanisms initiate at and largely affect the electrode–electrolyte interface.^15^ Thus, restricting the direct contact between the NMC811 cathode and electrolyte during the high state-of-charge (SOC) is essential to suppressing parasitic reactions and improving stability at higher cutoff voltages. One way to achieve this interface stability is through the development of a uniform conducting coating layer.^9,16^ To date, different coating materials have been investigated to modify Ni-rich NMC cathodes.^17,18^ Metal fluoride-based coatings have been shown to improve electrochemical performance due to their superior chemical stability compared with other chemical compositions.^19−23^ In fact, the main byproduct of the electrolyte salt (LiPF_6_) decomposition, LiF, is an integral component in the CEI layer.^24^ It is chemically stable in the electrolyte environment and resistant against hydrofluoric acid (HF) that prevents cathode material degradation during the cycling period. It has a wide electrochemical stability between 0–6.4 V vs Li/Li^+^ which is critical in high-voltage cycling conditions. These properties make LiF an effective coating for addressing the instability issue at the electrode–electrolyte interface.^12,25−29^ As a preformed artificial CEI layer, the coating intends to mimic the natural LiF component developed during the initial formation cycles in LIB operation. In this way, a stable interface is established prior to cycling and the undesirable sacrifice of electrolyte and active material during irreversible side reactions is reduced.^27^

The application of LiF as a surface coating has been demonstrated in a number of cathode materials, including LiCoO_2_,^30^ NMC111,^31^ and NMC811.^26,29^ These studies performed such surface modification by the wet chemistry method, which is known to encounter difficulties in terms of controlling the coating layer thickness, surface coverage, and uniformity.^32^ If not managed well, these coating parameters can significantly affect the charge transfer at the electrode–electrolyte interface and, consequently, limit the cathode material performance.^33−35^ In order to circumvent these challenges, atomic layer deposition (ALD) was adapted in this work to develop a nanoscale uniform LiF coating. ALD is a thin film deposition technique that uses alternating surface reactions between a substrate and gaseous precursors. The sequential and self-terminating surface reactions between the substrate and precursors limit the deposition to one atomic layer per cycle. Thus, the method enables control of coating thickness at the nanometer level and more uniform surface coverage compared with traditional wet chemistry methods.^33−37^ This is particularly beneficial in this work as the main concern in utilizing LiF as a coating material is its poor Li^+^ conductivity.^28,38^ The ability of ALD to control the coating thickness and uniformity allows the development of a stable coating without significantly impeding charge transfer. Furthermore, another advantage of ALD is its conformal deposition mechanism, which allows coating a wide range of substrates.^32,34,35^ Hence, the direct deposition of LiF on the composite electrode substrate as an artificial CEI layer rather than the active cathode material is achievable through this method.

The development of metal fluoride-based coatings via ALD for LIB applications is still at its infancy, compared with other materials such as metal oxides.^11,19,22,34^ Mäntymäki et al.^39,40^ reported the first successful preparation of LiF by ALD. The studies mainly focused on the optimization of the deposition method and the characterization of the LiF thin film on a Si wafer substrate. Chen et al.^41^ studied ALD LiF on Li metal and reported enhanced electrochemical stability by inhibiting dendrite formation. So far, the application of LiF by ALD on cathode materials has only been investigated on LiMn_1.5_Ni_0.5_O_4_ (LMNO) powder^25^ and NMC811 electrode.^28^ Tiurin et al.^25^ deposited LiF on LMNO powder using two different fluorine sources. Both coatings improved the stability of LMNO when cycled at 0.1C between 3.5 and 4.85 V vs Li/Li^+^. Xie et al.^28^ reported the deposition of LiF, LiAlF_4_, and AlF_3_ on the NMC811 electrode, but the study primarily investigated the cycling stability of LiAlF_4_-coated NMC811 at 2.75–4.5 V vs Li/Li^+^. Interestingly, the rate performance showed significantly lower specific discharge capacities of LiF-coated NMC811 compared with the bare sample.^28^ To our knowledge, there is currently no reported study that focuses on the investigation of electrode–electrolyte interface stability during high voltage cycling of an NMC811 electrode with preformed LiF via ALD. The influence of the artificial CEI layer on the electrode–electrolyte interface growth during prolonged cycling remains to be evaluated.

In this work, we report for the first time the high voltage cycling stability of an ALD LiF-coated NMC811 electrode in half-cell (4.6 V vs Li/Li^+^) and full-cell (4.5 V vs graphite) configurations. As capacity loading is an integral part in the energy density and cost reduction, the study utilizes a moderately high loading of NMC811 active material. The degree of irreversible parasitic reactions impacting the capacity retention is compared for uncoated NMC811 and LiF-NMC811. In addition, the influence of LiF on the NMC811 structural stability is evaluated by post-mortem analysis of the cycled electrodes. As LiF is an intrinsic compound in the CEI, the key findings from the structural and chemical characterization of the electrodes harvested from half-cell and full-cell can provide new insights on the structural and interfacial stability of NMC811 electrodes with artificially formed CEI. Additionally, this work addresses a gap in fluoride-based cathode coatings developed by ALD.

## 2 Experimental Section

### 2.1 Active Material Synthesis and Electrode Preparation

The NMC811 active cathode material was prepared via a solid-state reaction of the Ni_0.8_Mn_0.1_Co_0.1_(OH)_2_ precursor (Umicore Finland Oy) and 5% excess LiOH (Sigma-Aldrich, 98%). The materials were thoroughly mixed and then heated at 800 °C in a tube furnace (Nabertherm) for 12 h under an O_2_ atmosphere. To fabricate positive electrodes, an electrode slurry was mixed using a dispergator (Dispermat) at 500 rpm and then coated on 20 μm thick aluminum foil (MTI). The dry content of the positive electrode slurry is composed of 95 wt % NMC811 active material, 3 wt % conducting carbon black (Timcal Super C65), and 2 wt % polyvinylidene fluoride (PVDF, Solvay, Sole 5130) binder dissolved in *N*-methyl-2-pyrrolidone (NMP, Alfa Aesar) as the solvent. The printed electrode was dried in a fume hood overnight and subsequently in an oven at 80 °C for 4 h. Afterward, circular electrodes with a diameter of 14 mm for the coin cell configuration and 18 mm for the three-electrode cell configuration were cut. The prepared electrode had a loading of 10–10.5 mg cm^–2^, which corresponds to ∼2 mA h cm^–2^ based on 200 mA h g^–1^ for NMC811.

### 2.2 LiF Coating via ALD

LiF coating depositions were carried out in a flow-type hot-wall ALD reactor (F-120, ASM Microchemistry) at 220 °C. Lithium *tert*-butoxide (LTB, Strem, 98+%) and titanium(IV) fluoride (TiF_4_, Sigma-Aldrich) were used as precursors. The LTB and TiF_4_ powders were placed inside the reactor in open boats heated to 170–180 °C and 120–130 °C, respectively. N_2_ (99.999%) was used as carrier and purge gas, and reactor pressure was kept at 4.5–6 mbar. The pulse lengths of LTB and TiF_4_ were set at 2 s, separated by 15 s of purging period. Initial depositions were done on a 1.5 × 1.5 cm^2^ Si wafer to optimize the deposition parameters and coating thickness. Afterward, the LiF coating was deposited on the prepared NMC811 electrodes using 150, 200, and 250 ALD cycles.

### 2.3 Structural and Chemical Characterization

The thickness of the LiF coating deposited on the Si substrate was measured by X-ray reflectivity (XRR, PANalytical X’Pert Pro Alpha 1 MPD) using Cu K_α1_ radiation at 45 kV and 40 mA. The XRR data were fitted using the PANalytical X’Pert reflectivity program, and the fitting procedure is described in the work of Ahaliabadeh et al.^42^ To confirm the successful deposition of LiF and its effect on the NMC811 electrode, different structural and chemical characterization techniques were employed. X-ray diffraction (XRD, PANalytical X’Pert Pro Alpha 1) data were obtained in a 2θ range of 10–70° using Cu K_α1_ radiation operated at 45 kV and 40 mA to determine the crystalline structure. The particle size and morphology of the uncoated NMC811 and LiF-coated NMC811 electrodes were evaluated by using a scanning electron microscope (SEM, JEOL, JIB-4700F) set at an operation voltage of 5 kV. The SEM is equipped with an energy dispersive X-ray spectroscopy (EDS) detector operated at 10 kV for elemental distribution analysis. XRD and SEM–EDS were also performed on the NMC811 powder active material prior to electrode slurry preparation. Surface elemental composition was studied by X-ray photoelectron spectroscopy (XPS, Kratox Axis Ultra) equipped with a monochromatic Al Kα X-ray source. High-resolution scans were carried out using a 20 eV pass energy at a 0.1 eV energy step. The XPS spectra were calibrated using the C–C signal at a binding energy of 284.8 eV. The cross-sectional structure and morphology of the LiF-coated NMC811 were investigated by transmission electron microscopy (TEM), carried out with a double aberration-corrected microscope (JEOL, JEM-2200FS) operated at 200 kV. The elemental composition was measured using a TEM-integrated energy dispersive spectroscopy (EDS) detector. The cross-sectional TEM lamellas were prepared by focused ion beam (FIB) milling on a JEOL JIB-4700F microscope with a Ga ion source. Prior to milling, a ∼1.5 μm-thick Pt layer was deposited on the surface of a desired particle to protect it from being damaged by an ion beam during processing. Ga ion beam energy/probe current were applied with 30 kV/10 nA and 5 kV/30 nA for coarse and fine millings, respectively.

### 2.4 Electrochemical Cell Assembly and Testing Protocols

The uncoated NMC811 and LiF-coated (LiF-NMC811) electrodes were calendered and then dried at 80 °C for at least 12 h under vacuum before being transferred to an Ar-filled glovebox (Jacomex, <1 ppm of H_2_O and O_2_). CR2016—type coin cells (Hohsen) were fabricated in half-cell and full-cell configurations using uncoated NMC811 and LiF-NMC811 as the cathode. The half-cell set up was assembled with the 14 mm diameter cathode, 19 mm diameter lithium–metal foil (MSE Supplies), and 19 mm diameter glass fiber (Whatman GF/A) separator soaked in 200 μL of 1 M lithium hexafluorophosphate (LiPF_6_) dissolved in 1:1 (v/v %) ethylene carbonate (EC) and dimethyl carbonate (DMC) (BASF) solution as an electrolyte. The full-cell comprised 14 mm diameter of cathode, 18 mm diameter of graphite negative electrode, and a 19 mm polyolefin separator soaked in 13 wt % LiPF_6_ in 20:25:40 wt % EC/DMC/EMC (ethyl methyl carbonate) with a 2 wt % vinylene carbonate (VC) solution. Capacity balancing of anode and cathode (N/P ratio) was set to ∼1.3–1.4:1. Three-electrode cells (EL-CELL, ECC-ref) were assembled using an 18 mm cathode, lithium–metal foil (MSE Supplies) as the counter and reference electrodes, and a 1.5 mm thick glass fiber separator soaked in 500 μL of 1 M LiPF_6_ in a 1:1 (v/v %) EC/DMC solution. All coin cells and EL-Cells were allowed to rest for 24 h prior to electrochemical testing.

The assembled half-cells and full-cells were tested by galvanostatic charge–discharge tests (Land Cycler) at 1C in the potential range of 3.0–4.6 V vs Li/Li^+^ and 2.8–4.5 V vs graphite at room temperature. A rate capability test was performed on a half-cell by constant-current charging at 0.2C, followed by constant-voltage (CC–CV) charging at 4.6 V with a 0.03C current limit. Constant-current (CC) discharging was conducted at different current densities, ranging from 0.1 to 5C. Prior to the tests, formation cycles were performed. For the half-cell, one charge–discharge cycle at 0.03C and three charge–discharge cycles at 0.1C from 3.0 to 4.3 V vs Li/Li^+^ were conducted as the activation process. For a full-cell, five charge–discharge cycles at 0.1C between 2.9 and 4.2 V vs graphite were done. At least two parallel samples were tested to confirm the results.

Cyclic voltammetry (CV) and electrochemical impedance spectroscopy (EIS) tests were performed on the three-electrode cell by using a BioLogic potentiostat (MPG-205). The CV measurements were performed between 3.0 and 4.6 V vs Li/Li^+^ at 0.02 mV s^–1^ after the initial formation cycles and after the 100th charge and discharge cycle at 1C. The impedance spectra were obtained at 3.9 V over the frequency range of 100 kHz and 10 mHz preceding the 1st cycle and after the 25th, 50th, and 100th cycles at 1C. ZView software was used for spectral fitting.

### 2.5 Post-Mortem Characterization

Cycled uncoated NMC811 and LiF-NMC811 cathodes and graphite anodes were retrieved from disassembled cells for structural and chemical characterization. Prior to the measurements, the electrodes were washed with DMC and dried overnight inside the glovebox.

## 3 Results and Discussion

### 3.1 Structural and Chemical Characterization

**Figure 1:**
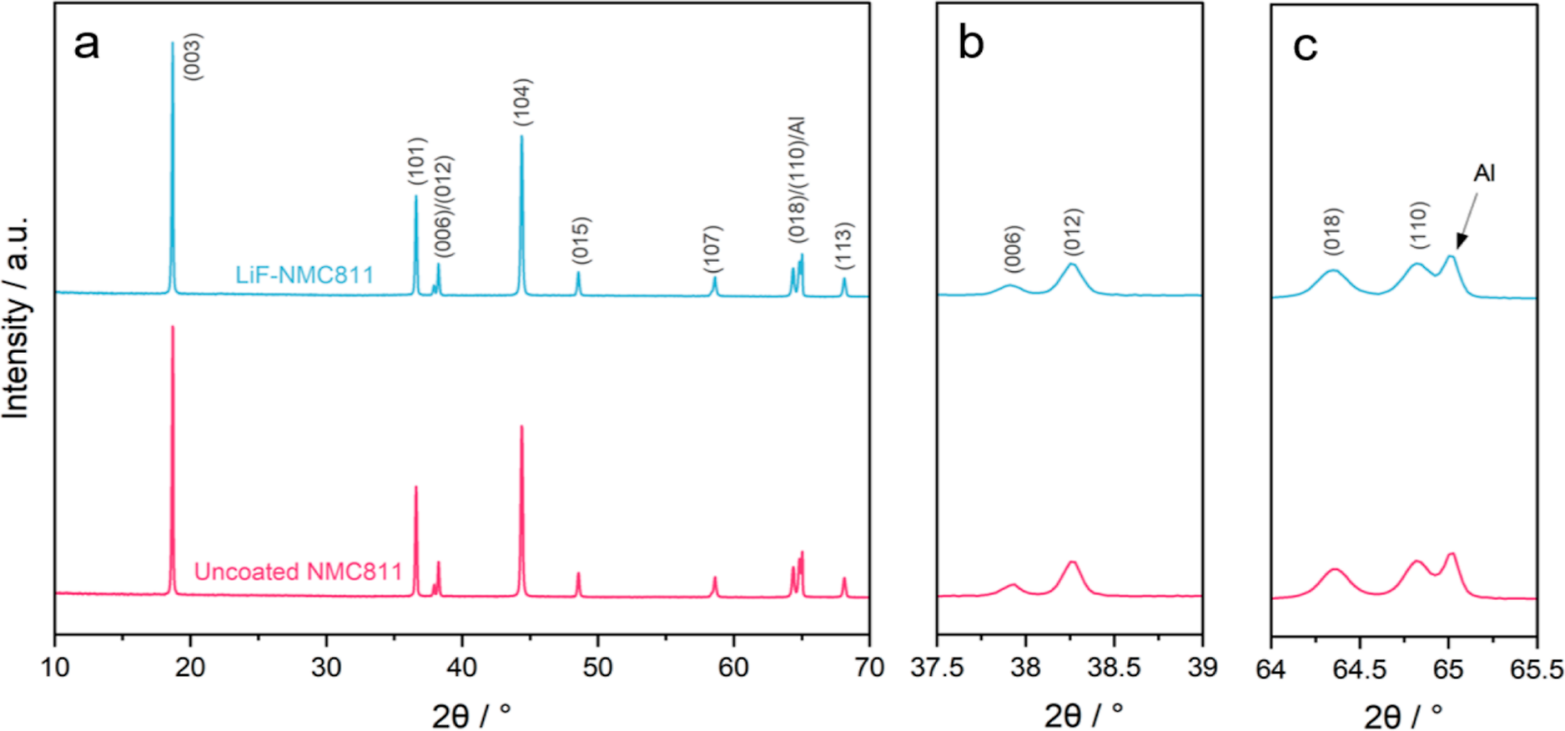
XRD patterns of (a) the uncoated NMC811 and LiF-NMC811 electrodes and the corresponding magnified views for splitting of the (b) (006)/(012) and (c) (018)/(110) peaks.

Details of the NMC811 powder active material characterization prior to electrode slurry preparation are shown in Figure S1 (Supporting Information). The diffraction patterns of uncoated NMC811 and LiF-NMC811 electrodes, shown in Figure 1a, demonstrate similar peaks exhibiting α-NaFeO_2_ structure with the *R*3̅*m* space group.^43−45^ A closer look at the distinct splitting of the (006)/(012) and (018)/(110) peaks (Figure 1b,c, respectively) confirms the well-ordered layered structure of the samples.^46^ Additionally, the intensity ratio *I*_(003)_/*I*_(004)_ is 1.56 for both samples, which is above the acceptable cation mixing value of 1.2, signifying a low degree of Ni^2+^/Li^+^ mixing.^12,47,48^ Apart from the Al current collector peak observed at 65°, no LiF-related peak is visible in the coated electrode, which may be due to the minimal quantity of the nanoscale coating or its amorphous nature. Similar findings were reported by previous studies on NMC811 coated with LiF.^26,29,31,49^ The XRD analysis confirms that the addition of the LiF coating does not affect the NMC811 bulk structure.

**Figure 2:**
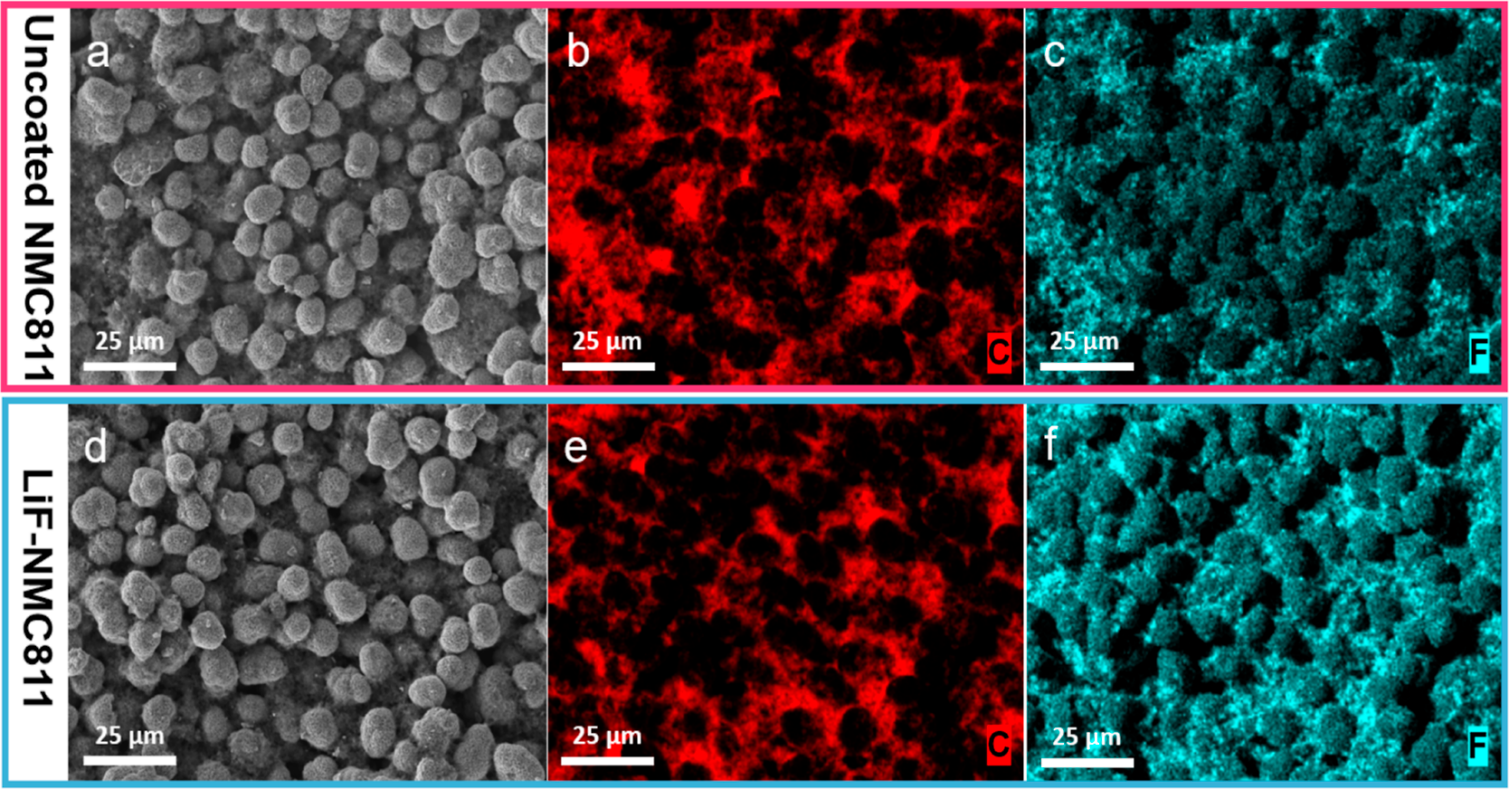
SEM images and corresponding elemental maps of C and F for (a–c) the uncoated NMC811 and (d–f) the LiF-NMC811 electrodes.

Figure 2a shows the surface morphology of the NMC811 electrode prior to the LiF coating. The quasi-spherical secondary particles averaging 8–10 μm in diameter are dispersed in a porous binder-conducting carbon matrix, as confirmed by the distribution of C and F in Figure 2b,c, respectively. After coating, the LiF-NMC811 electrode retained a similar particle morphology and binder-conducting carbon matrix, as shown in Figure 2d–f. The elemental mapping of Ni, Mn, Co, and O (Figure S2 in Supporting Information) also demonstrates a homogeneous distribution before and after the coating, with no significant difference in the transition metal content. No evident coating layer can be observed on the surface, which may be due to its nanosized thickness. Nevertheless, the SEM–EDS results indicate that the LiF coating process does not affect the morphology of the bulk NMC811.

**Figure 3:**
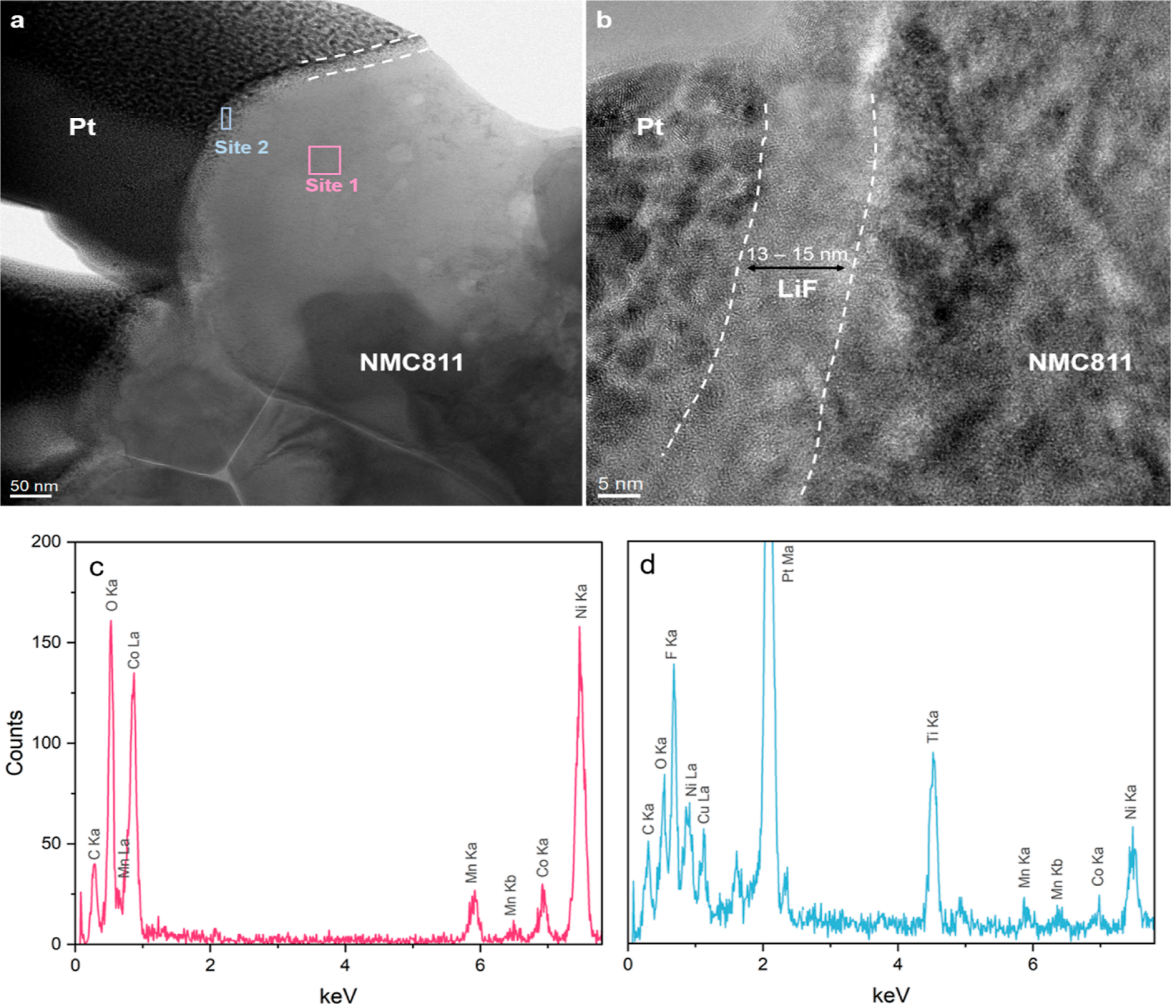
(a) Cross-sectional TEM image of NMC811 coated with LiF for 150 ALD cycles; (b) HR-TEM image of the NMC811 bulk and LiF-coating interface; and EDS spectra of (c) the NMC811 bulk structure (site 1) and (d) the LiF-coating layer (site 2).

To investigate the thickness and uniformity of the coating, cross-sectional TEM analysis was conducted on the LiF-NMC811 electrode prepared by using 150 ALD cycles. Figure 3a shows an overview TEM image composed of a dark region attributed to the deposited Pt during the lamella preparation, the bulk NMC811, and a distinct amorphous layer (marked by dashed white lines) between the Pt and NMC811 regions. The HR-TEM image of the interface (Figure 3b) shows an amorphous structure of the LiF coating with a thickness between 13 and 15 nm. Moreover, the HR-TEM image exhibits that a highly uniform surface coating is achieved via the ALD technique.

The EDS spectrum of the bulk (site 1) shown in Figure 3c displays peaks from NMC811. Due to the nanoscale thickness, the EDS mapping boundary of the coating layer (site 2) extends to the Pt and NMC811 regions, which explains their corresponding peaks in Figure 3d. Nevertheless, the presence of an F peak confirms the LiF coating on the surface. A Ti peak obtained from the TiF_4_ precursor used for the ALD coating is also observed.^25^ Mäntymäki et al.^39,40^ successfully deposited on Si wafer via ALD without Ti by using higher deposition temperatures (250–350 °C). However, in this work, the temperature has been limited to 220 °C to avoid the decomposition of the PVDF binder in the electrode. Xie et al.^28^ coated NMC811 electrodes with LiAlF_4_ using the same ALD precursors and reported minimal Ti impurity, with no detrimental effects on the electrochemical performance. Hence, the electrodes are deemed appropriate for further characterization. Overall, the TEM–EDS results confirm the successful coating of LiF on the NMC811 electrode and support the XRD findings regarding the amorphous structure of the coating.

**Figure 4:**
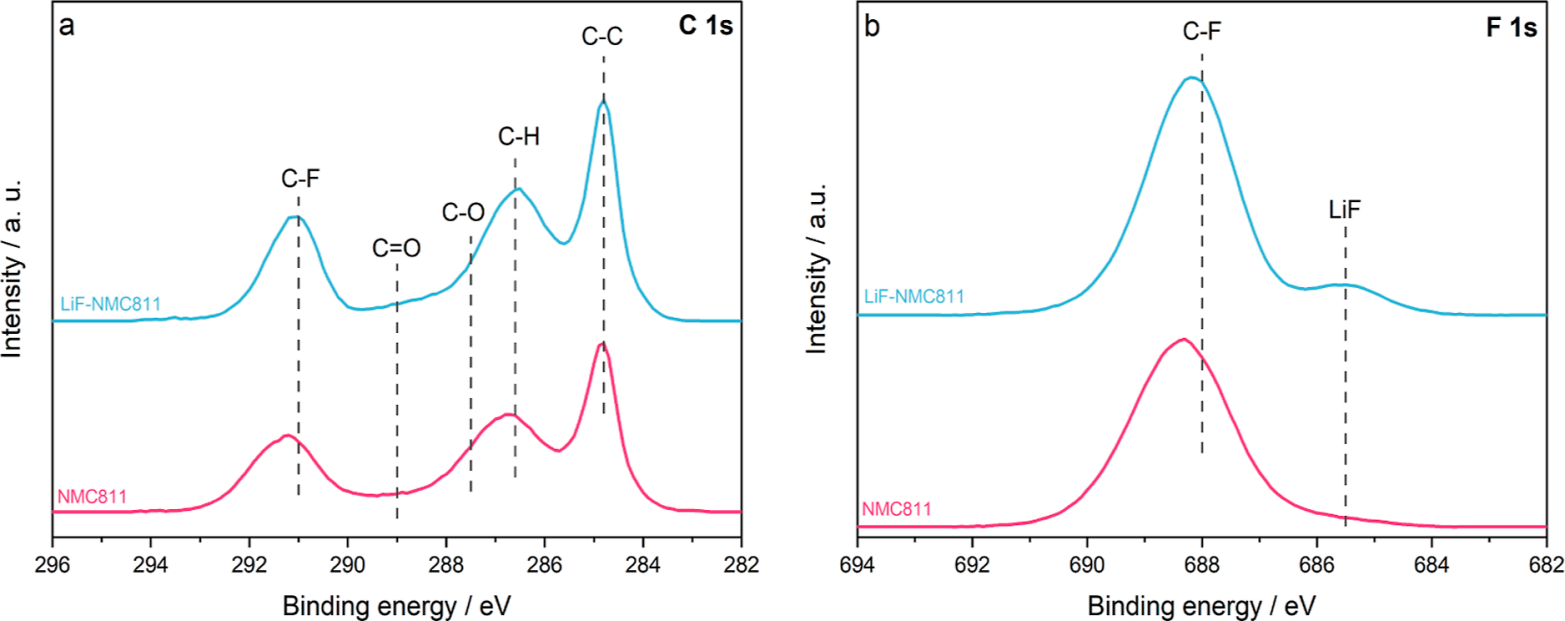
High-resolution XPS spectra of the uncoated NMC811 and LiF-NMC811 electrodes for (a) C 1s and (b) F 1s.

XPS analysis was performed to complement the TEM results by investigating the difference in surface chemistry of the uncoated NMC811 and LiF-NMC811 electrodes. The high-resolution spectra of C 1s and F 1s prior to cycling are shown in Figure 4a,b, respectively. Both samples show similar peaks for C 1s; C–C peak (∼284.8 eV) is assigned to carbon black, while the C–H (∼286.6 eV) and C–F (∼291 eV) peaks are attributed to the PVDF binder.^3,50−52^ The structure at ∼289 eV corresponds to C=O and can be due to exposure of the electrode to air during storage.^3,10^ In F 1s, the C–F peak (∼688 eV) is related to the PVDF binder and corresponds to the C–F peak in C 1s.^51,52^ However, a bump at ∼685.5 eV attributed to Li–F is visible for LiF-NMC811.^50^ This is in line with the TEM findings and further confirms the presence of the LiF coating on the NMC811 electrode. A similar confirmatory peak is found in different studies on LiF coatings on cathode materials, both for powders and electrodes.^19,25,28−30^

### 3.2 Cycling Stability in Half-Cell Configuration

One of the advantages of using the ALD technique to develop the coating is its ability to tailor the film thickness by adjusting the number of deposition cycles.^33^ For the LiF coatings deposited on the Si wafer substrate, XRR analysis reported a growth per cycle (GPC) value of 0.07 nm. This is comparable to the coating thickness on the NMC811 electrode substrate with 150 ALD cycles measured by TEM in Section 3.1. The slight variation may be due to the difference in substrate material, which can affect the film growth rate during the ALD process.^27^ To determine the optimal coating thickness, a preliminary cycling test was conducted for the NMC811 electrodes coated with LiF using a different number of ALD cycles. As shown in Figure S3 (Supporting Information), the coated samples deliver comparable capacity by the end of 100 cycles, which is all higher than the uncoated NMC811. However, the specific discharge capacity decreases as the number of ALD cycles increases. This demonstrates that the thickness of the LiF coating plays a critical role in the electrochemical performance of the NMC811 electrode, and the use of ALD in enabling precise control of the coating thickness is beneficial in this regard. LiF-NMC811 with 150 ALD cycles delivers the highest specific discharge capacity and capacity retention after 100 cycles; this sample is then used for further electrochemical performance assessment.

**Figure 5:**
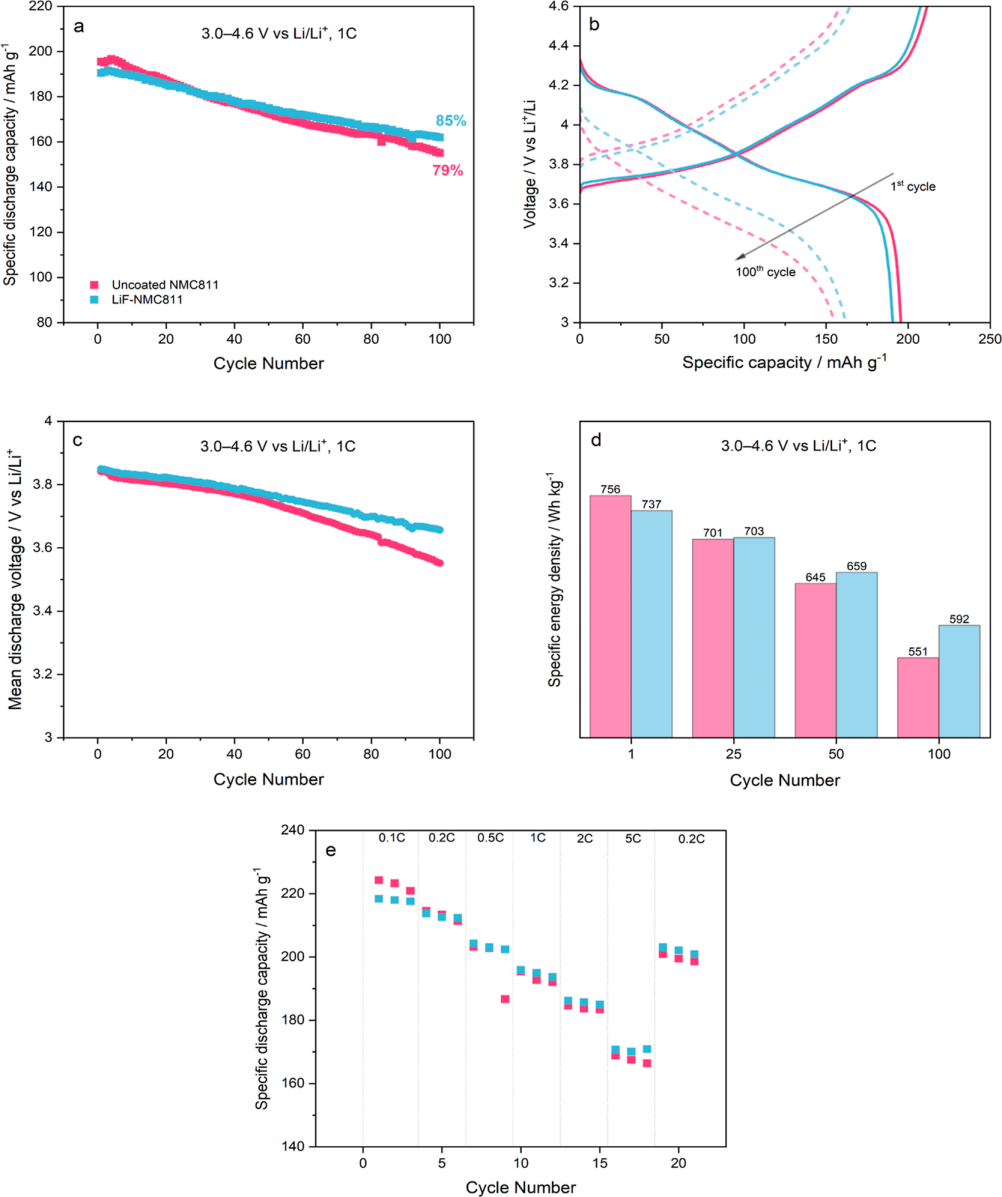
Comparison of (a) cycling performance over 100 cycles at 1C; (b) charge–discharge profile of 1st and 100th cycles at 1C; (c) mean discharge voltage over 100 cycles at 1C; (d) specific energy density at the 1st, 25th, 50th, and 100th cycles; and (e) rate performance for three cycles at 0.1 to 5C of uncoated NMC811∥Li (pink) and LiF-NMC811∥Li (blue).

The influence of the LiF coating on the high voltage (3.0–4.6 V vs Li/Li^+^) cycling stability of NMC811 in the half-cell configuration is presented in Figure 5. The specific discharge capacity behavior over 100 charge/discharge cycles at 1C is shown in Figure 5a. After the formation cycles, the discharge capacities are 196 ± 0.4 and 191 ± 0.5 mA h g^–1^ for uncoated NMC811 and LiF-NMC811, respectively. The lower value for LiF-NMC811 is due to the presence of the coating, which serves as an additional layer to the Li^+^ diffusion pathway.^22,25,43,53^ Consequently, the specific capacity–voltage profile (Figure 5b) of LiF-NMC811 exhibits a slightly higher overpotential in the first charge/discharge cycle. By the end of 100 cycles, LiF-NMC811 delivers a higher capacity retention of 85 ± 0.6% (161 ± 0.7 mA h g^–1^) compared with uncoated NMC811 with 79 ± 0.3% (155 ± 0.3 mA h g^–1^). The evolution of the specific capacity–voltage profiles by the 100th cycle supports this capacity-fading behavior as the uncoated NMC811 charge/discharge curves experience a bigger shift in overpotential compared with the LiF-NMC811. The improved cycling stability of LiF-NMC811 can be attributed to the presence of a coating that blocks the contact between the highly delithiated cathode and the electrolyte, thereby limiting harmful side reactions that trigger further growth of the cathode-electrolyte interface (CEI) layer.^12,28,48^ Li et al.^54^ studied the failure mechanism of NMC811∥Li cells and reported that the more pronounced electrolyte decomposition is the main cause of poor cycling performance above 4.2 V vs Li/Li^+^. Despite this impediment at high cutoff voltages, the capacity retention for LiF-NMC811 in this work is comparable to studies that utilized a narrower cycling window.^21,29,43,45,55^ The benefit of preforming LiF on the NMC811 surface to curb material degradation during prolonged cycling is further discussed in the CV and EIS analyses.

Figure 5c presents a gradual and more stable decline of the mean discharge voltage curve for LiF-NMC811 as a result of a slower polarization growth. With lesser voltage losses over the cycling period, the LiF-NMC811∥Li cell exhibits a better retention in specific energy density (592 ± 1.9 W h kg^–1^) than the uncoated NMC811∥Li (551 ± 8.9 W h kg^–1^) cell, as shown in Figure 5d. The impact of the LiF coating on the rate performance of the NMC811 electrode is also evaluated using CC–CV charge and CC discharge protocols at different C rates. At 0.1C, uncoated NMC811 and LiF-NMC811 can deliver specific discharge capacities of 223 ± 2.1 and 218 ± 1.3 mA h g^–1^, respectively. Discharge capacities at 0.2 and 0.5C are comparable; however, uncoated NMC811 starts to decline faster at higher C rates compared with LiF-NMC811. The hold at high voltages during the CC–CV charging reduces the Li^+^ concentration gradient between the surface and the bulk, leading to improved kinetics.^56^ However, parasitic side reactions at the electrode–electrolyte interface are exacerbated by the high voltage hold, resulting in poor capacity retention for uncoated NMC811.^54,57^ Jiang et al.^20^ reported a similar trend in rate performance for LiF–LaF_3_-coated NMC811 evaluated at the same cutoff voltage. After cycling at high C rates (2 and 5C), uncoated NMC811 and LiF-NMC811 deliver specific discharge capacities of 200 ± 2.1 and 202 ± 0.5 mA h g^–1^, respectively, when the current density is shifted back to 0.2C. The higher capacity retention of LiF-NMC811 (94.9 ± 0.5%) in comparison with uncoated NMC811 (93.7 ± 0.5%) is a good indicator of the structural stability brought by the coating even during rapid Li^+^ intercalation/deintercalation. Despite LiF being reported as a coating material with relatively low ionic conductivity,^28,41^ these results show that the coating does not have a detrimental effect on the rate capability of NMC811 and highlight the advantage of controlling the thickness and uniformity of the coating layer via ALD.^38,41^ Overall, the specific discharge capacity, capacity retention, and specific energy density values presented in this work prove LiF as an effective electrode coating for cycling at a high cutoff voltage.

**Figure 6:**
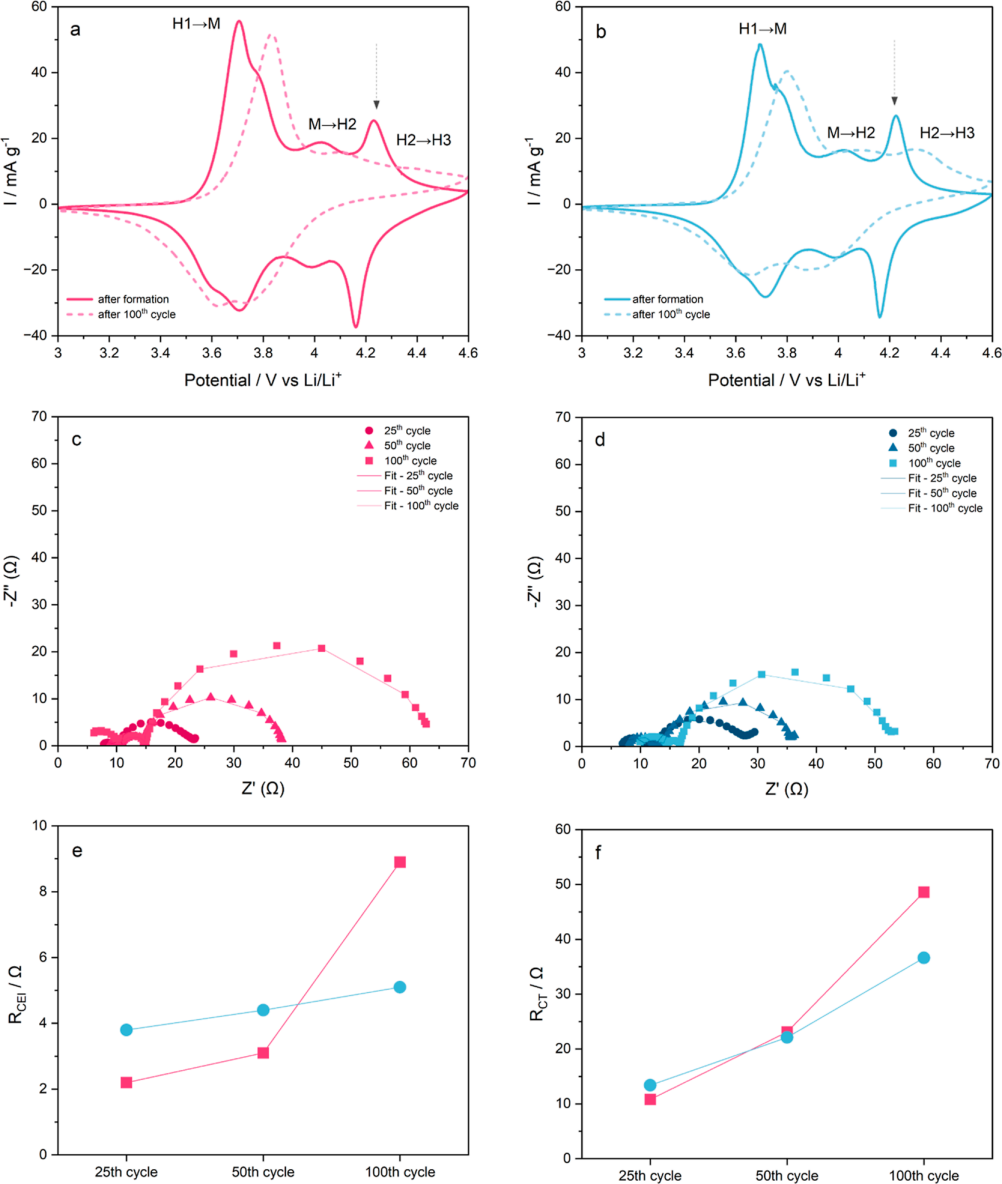
Comparison of (a,b) CV curves measured after initial formation cycles and after 100 cycles at 0.02 mV s^–1^; (c,d) Nyquist plots at 25th, 50th, and 100th cycles; and fitting results of impedance spectra for (e) *R*_CEI_ and (f) *R*_CT_ of uncoated NMC811 (pink) and LiF-NMC811 (blue).

CV curves of uncoated NMC811 and LiF-NMC811 measured between 3.0 and 4.6 V versus Li/Li^+^ are shown in Figure 6a,b, respectively. After the initial formation cycles, both samples show three distinct pairs of anodic/cathodic peaks assigned to the multiphase transition of a Ni-rich layered oxide cathode material. In the anodic scan, the first peak is assigned to the transition from a hexagonal (H1) phase to a monoclinic (M) phase, followed by a second peak for the transition to another hexagonal (H2) phase, and then a third peak for the transition to the hexagonal (H3) phase.^16,44,57−60^ After 100 charge/discharge cycles, the samples experience a higher degree of polarization, indicated by the shift of oxidation and reduction peaks to higher and lower potentials, respectively.^59^ Additionally, the intensity reduction of the anodic/cathodic peaks signifies capacity decline, in line with the cycling performance in Figure 5. The root cause of these behaviors can be traced back to the detrimental effect of high-cutoff voltage cycling. The H2–H3 phase transition, which appears above ∼4.24 V, represents the unit cell contraction in the *c*-axis during high SOC.^20,26,45^ This lattice parameter describes the distance between the Li slabs upon lithiation/delithiation.^2,12,61^ A study by Ryu et al.^62^ highlights the damaging effect of repeated H2–H3 transitions to the lattice structure. The anisotropic volume changes may lead to the intergranular cracking of secondary particles and create new sites for CEI layer formation.^2,56^ Moreover, the formation of the NiO rock-salt phase is more susceptible to occurring in a highly delithiated Ni-rich NMC due to cation mixing and oxygen loss during cycling.^7,13,55^ The presence of NiO on the surface slows down the Li^+^ mobility due to its poor ionic conductivity, thereby increasing cell impedance.^60,63^ The disappearance of the H2–H3 peak for uncoated NMC811 after 100 cycles suggests an irreversible NiO rock-salt phase transition on the surface.^16^ In contrast, LiF-NMC811 continues to exhibit the H2–H3 transition even after the 100th cycle, showing less impedance buildup as evidenced by its higher capacity retention. Limiting the cutoff voltage to 4.1 V versus Li/Li^+^ avoids the detrimental H2–H3 region but, in turn, significantly lowers the discharge capacity and energy density of a Ni-rich layered oxide material.

The impedance evolution of LiF-NMC811 reveals that the enhanced capacity retention is mainly due to the development of a more stable artificial CEI layer in the form of a LiF coating. The Nyquist plots of uncoated NMC811 and LiF-NMC811 at the 25th, 50th, and 100th cycles are presented in Figure 6c,d, respectively. The EIS spectra are composed of five parts, which include: (1) an intercept in the real axis at the high frequency region related to the ohmic resistance (*R*_S_); (2) a semicircle related to the Li^+^ diffusion resistance through the CEI layer (*R*_CEI_); (3) a semicircle at the intermediate frequency region related to grain boundary resistance (*R*_GB_); (4) a semicircle at the low frequency region related to the charge transfer resistance at the electrode/electrolyte interface (*R*_CT_); and (5) a sloping line at the low frequency region related to the Li^+^ diffusion through the bulk electrode.^7,28,42,63^ Based on the equivalent circuit shown in Figure S4 (Supporting Information), the impedance spectra were fitted, and the *R*_CEI_ and *R*_CT_ values are shown in Figure 6e,f, respectively.

LiF-NMC811 reports higher *R*_CEI_ (3.6 Ω vs 1.5 Ω) and *R*_CT_ (4.5 Ω vs 2.2 Ω) impedance values compared with uncoated NMC811 preceding the first charge–discharge cycle at 1C, as shown in the Nyquist plots in Figure S6 (Supporting Information). The ALD-formed LiF coating on the NMC811 electrode surface serves as an added barrier to the Li^+^ mobility, thereby causing additional internal resistance.^53^ This supports the lower specific discharge capacity and higher overpotential displayed by LiF-NMC811 during the first cycling at 1C in Figure 5a,b, respectively. Nonetheless, the extended cycling period demonstrates the advantage of a coated electrode as the *R*_CEI_ has a more stable increase from 4.4 Ω after 50 cycles to 5.1 Ω after 100 cycles for LiF-NMC811. On the other hand, *R*_CEI_ values for uncoated NMC811 significantly increase from 3.1 Ω after 50 cycles to 9.0 Ω after 100 cycles. This drastic increase is due to the continuous accumulation of byproducts from electrolyte decomposition and transition metal dissolution onto the CEI layer.^6,12^ Consequently, the continuous growth of the CEI layer affects the charge transfer movement across the interface during cycling.^56^ The slowdown of Li^+^ intercalation/deintercalation is more evident in the uncoated NMC811, as shown by the sharp increase of *R*_CT_ values. After 100 cycles, the *R*_CT_ of uncoated NMC811 is 48.8 Ω, almost five times higher than the 10.8 Ω value after 25 cycles. Meanwhile, LiF-NMC811 exhibits a smaller increase from 13.4 Ω after 25 cycles to 36.6 Ω after 100 cycles. Aside from the nonuniform growth of the CEI layer, the formation of a NiO rock-salt structure on the surface of uncoated NMC811, as confirmed by CV results, may have caused the slowdown of charge transfer reactions across the electrode–electrolyte interface.^55,62,64,65^ To corroborate the results from the initial cycling tests of the LiF-coated electrodes, the impedance evolution of the LiF 250 sample was also analyzed. As indicated in Figure S6 (Supporting Information), LiF 250 has a more stable increase of *R*_CEI_ and *R*_CT_ values compared with uncoated NMC811. However, its overall impedance is still higher compared to the LiF-NMC811 sample. Once again, this emphasizes the need to determine the optimum coating thickness via ALD. The EIS results show that preforming a stable coating such as LiF can lessen impedance buildup and improve cycling stability by effectively suppressing harmful side reactions at the interface, even at a high cutoff voltage.

### 3.3 Cycling Stability in Full-Cell Configuration

**Figure 7:**
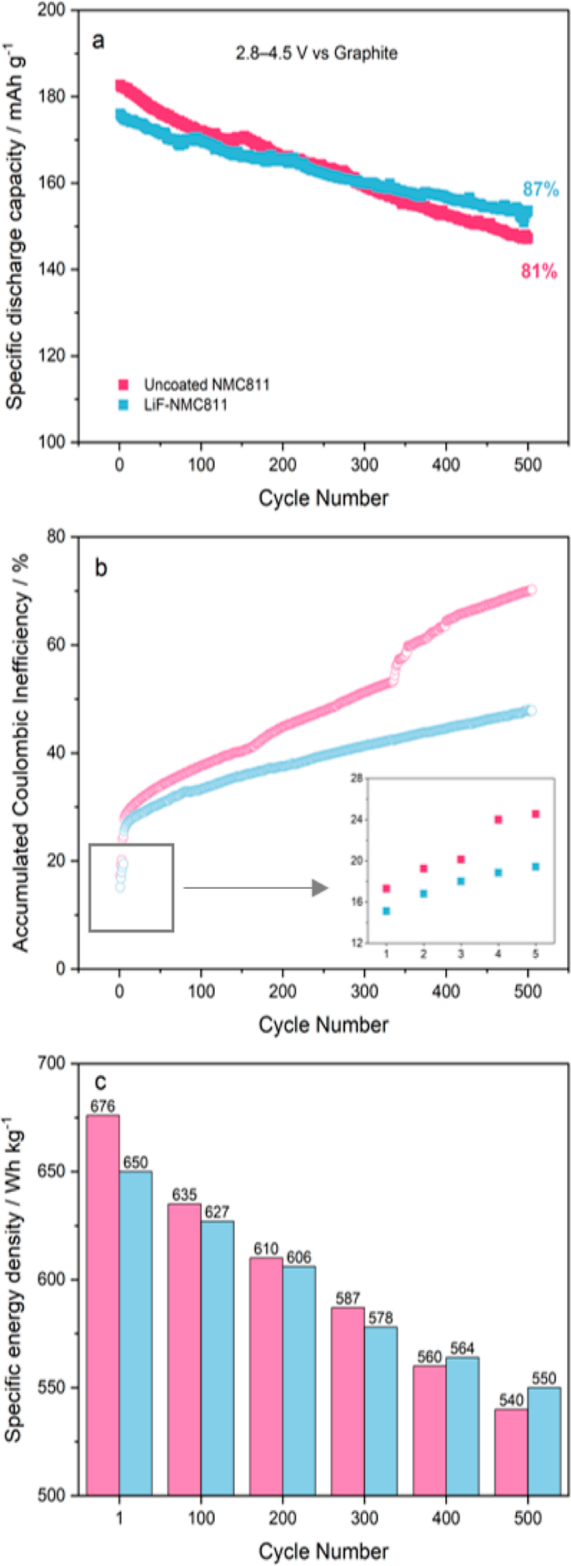
Comparison of the (a) long-term cycling performance over 500 cycles at 1C; (b) ACIE over 500 cycles at 1C (inset: first five formation cycles at 0.1C); and (c) specific energy density at the 1st, 100th, 200th, 300th, 400th, and 500th cycles of uncoated NMC811∥graphite (pink) and LiF-NMC811∥graphite (blue).

Aside from the practical application value of utilizing one of the leading anode materials in commercial LIBs, assembling full-cells with graphite can help evaluate the stabilizing effect of the LiF coating without the deleterious interaction between the electrolyte and Li metal.^1,66^ Moreover, the cutoff voltage (4.5 V vs graphite) employed in this work is higher compared with existing studies that investigated the cycling stability of an NMC811 cathode paired with a graphite anode.^13,57,67,68^Figure 7a compares the long-term cycling performance of the uncoated NMC811 and LiF-NMC811 in the full-cell configuration at 1C. Similar to the half-cell cycling results, uncoated NMC811 delivers a slightly higher specific discharge capacity of 183 ± 1.4 mA h g^–1^ compared with 176 ± 1.2 mA h g^–1^ for LiF-NMC811 at the initial cycle. After 500 charge/discharge cycles, the capacity retention for uncoated NMC811 is 80 ± 0.8% (147 ± 2.0 mA h g^–1^) while it is higher for LiF-NMC811 with 88 ± 1.2% (154 ± 1.5 mA h g^–1^). The lower initial specific discharge capacity in the full-cells, compared with the half-cells in Figure 5a, is primarily due to the consumption of active Li^+^ to form the solid electrolyte interphase (SEI) on the anode. During charging, some of the Li^+^ molecules that deintercalate from the NMC811 structure and intercalate into the layered graphite are irreversibly consumed in the SEI formation. Since the Li^+^ supply is not replenished by a Li metal source in a full cell configuration, a lower amount of Li^+^ returns to the positive electrode, resulting to a lower discharge capacity.^7,69^

The plot of accumulated Coulombic inefficiency (ACIE) reflects the magnitude of the side reactions during cycling, including electrolyte decomposition, CEI/SEI formation, TM dissolution, and active Li^+^ loss.^28,47^Figure 7b presents the ACIE plot during the long-term cycling at 1C, including the first five formation cycles at 0.1C (inset). Both samples show a high starting point due to the electrolyte and Li^+^ consumption for electrode–electrolyte interface formation. However, LiF-NMC811 demonstrates lower and more stable ACIE growth compared with uncoated NMC811, which is already 5% higher by the end of the formation cycles. This suggests that the LiF coating facilitates a more controlled development and growth of the CEI layer. Moreover, there is a sudden inflection point past 300 cycles for uncoated NMC811, which further demonstrates cycling instability. By the end of 500 cycles, LiF-NMC811 exhibits a significantly lower ACIE of 48 ± 5% compared with uncoated NMC811 with 70 ± 7%. The higher ACIE value of uncoated NMC811 implies that a fairly high amount of Li^+^ is irreversibly consumed by side reactions.^67,70^ The results show that the presence of the LiF coating improves the cycling performance of NMC811 by preventing active Li^+^ loss and impedance buildup.

Figure 7c compares the specific energy densities of uncoated NMC811∥graphite and LiF-NMC811∥graphite over 500 cycles at a 100-cycle interval. At the cell level, the use of graphite instead of Li metal as an anode reduced the energy density by 11% (1st cycle comparison with Figure 5d) at the initial cycle as expected.^1^ However, the advantage of using graphite as an anode can be clearly seen during long-term cycling. The energy densities of the full-cells at the 400th cycle are still comparable to the energy densities of the half-cells at the 100th cycle. The short cycle life of half-cells can be ascribed to the tendency of the Li metal anode to form dendrites.^71,72^ Nevertheless, it can be observed that further stability is provided by the LiF coating, which shows 550 ± 1.6 W h kg^–1^ energy density by the end of 500 cycles. Chen et al.^41^ coated Li metal with LiF via ALD and reported a similar improvement in Coulombic efficiency, even at the initial cycles.

### 3.4 Degradation Mechanism Analysis

**Figure 8:**
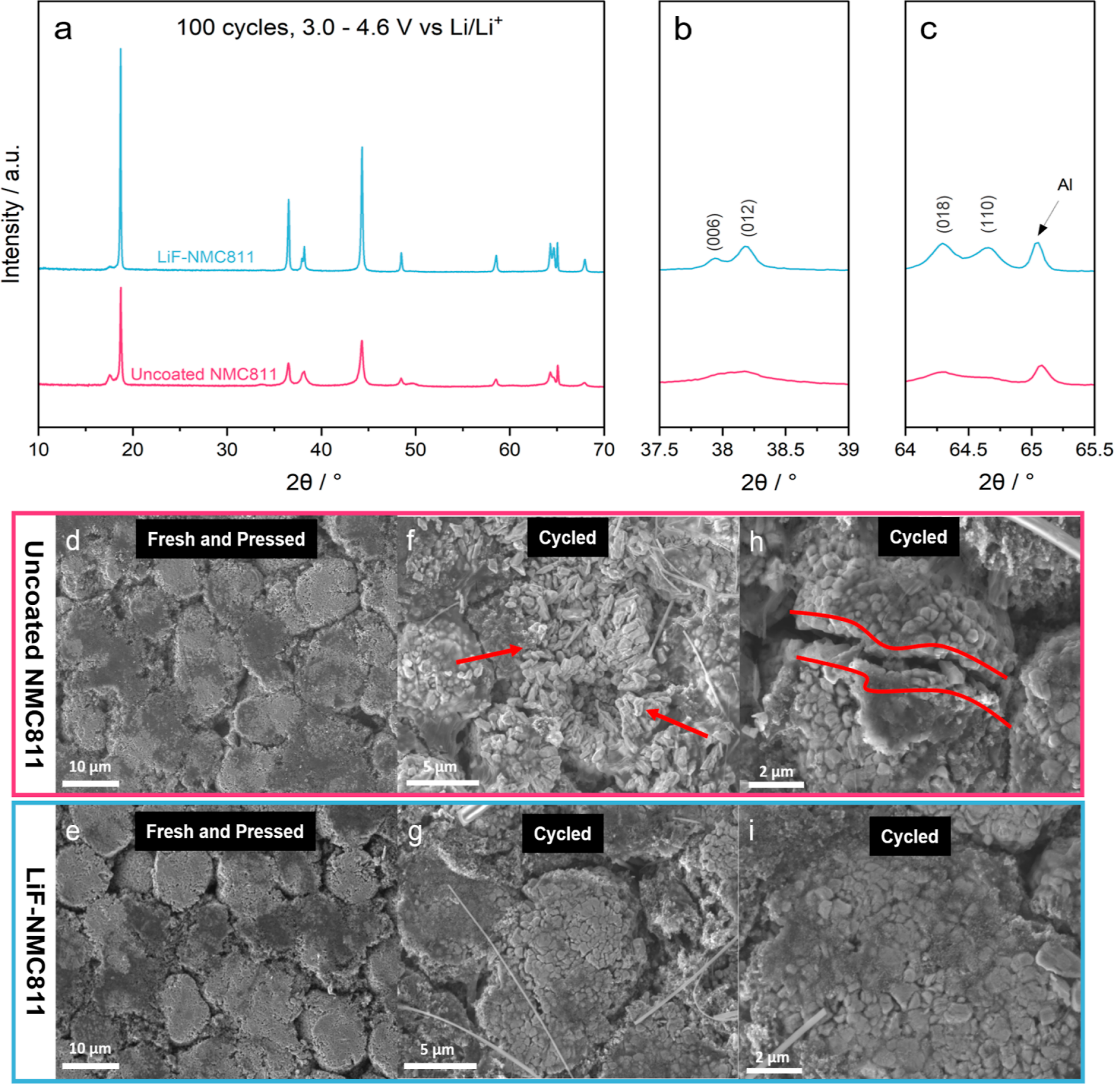
(a) XRD patterns of the uncoated NMC811 and LiF-NMC811 electrodes retrieved from the half-cells after 100 cycles at 1C; (b) magnified view of (006) and (012) peaks; (c) magnified view of (018) and (110) peaks; (d,e) SEM image of fresh and calendar-pressed uncoated NMC811 and LiF-NMC811 electrodes; (f,h) SEM images of the uncoated NMC811; and (g,i) LiF-NMC811 electrodes retrieved from the half-cells after 100 cycles at 1C.

To understand the underlying mechanism of the capacity fade, the structural and chemical changes on the cycled electrodes were investigated. Figure 8a shows the XRD patterns of the uncoated NMC811 and LiF-NMC811 electrodes retrieved from the half-cells after 100 cycles at 1C. There is a notable intensity reduction and broadening of the peaks for the cycled uncoated NMC811 electrode, indicative of more damage suffered by its layered structure compared to LiF-NMC811.^7^ Additionally, the indistinguishable splitting between the (006)/(012) peaks (Figure 8b) and the (018)/(110) peaks (Figure 8c) further confirms the absence of an ordered layered structure after substantial cycling.^46^ In contrast, the LiF-NMC811 electrode partially preserved the layered structure, which helped minimize the capacity fade.

Figure 8d,e shows that the calendared electrodes retained the quasi-spherical particle morphology. After cycling, the uncoated NMC811 electrode (Figure 8f,h) exhibits more fractured secondary particles with visible particle cracking and fragmentation. The fracture patterns are unlike the ones observed in Figure 8d. Thus, the damage is derived from the electrochemical cycling rather than the calendar-pressing process.^47^ Ryu et al.^62^ reported that the crack formation is caused by sporadic lattice expansion and contraction during cycling. Zhang et al.^73^ concluded that the main cause of crack formation is the fracturing of the NiO rock-salt layer as the repeated Li^+^ insertion and extraction during cycling introduced mechanical strain to the electrochemically inactive NiO phase. As seen in the CV results, uncoated NMC811 exhibits an H2–H3 transition at the initial cycles but eventually transforms to irreversible NiO. The combination of rapid volume change and mechanical strain to the fragile rock-salt structure led to the uncoated NMC811 secondary particle cracking and fragmentation. The collapsed and disconnected morphology not only broke off Li^+^ and electron pathways but also exposed fresh surfaces to the electrolyte for more side reactions to occur.^8^ This corroborates the EIS analyses, which report a higher increase in *R*_CEI_ and *R*_CT_ with extended cycling.^45^ In contrast, defects can hardly be observed on the surface of the LiF-NMC811 electrode, as the secondary particles are able to maintain the quasi-spherical morphology. Minimal cracks are present, but not to the same extent as those for the uncoated NMC811 electrode. Recent studies by Zhao et al.^71^ on NMC811 and Yang et al.^24^ on LiCoO_2_ reported enhanced structural integrity of the cathode materials brought about by the presence of an artificial CEI layer, which helps release stress accumulated in the structure.

**Figure 9:**
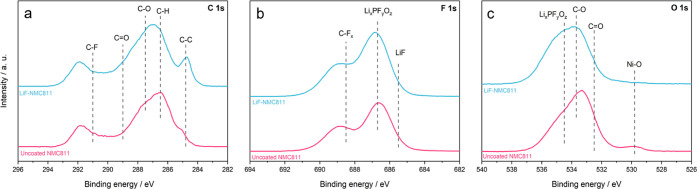
High-resolution XPS spectra of uncoated NMC811 and LiF-NMC811 electrodes retrieved from half-cells after 100 cycles at 1C: (a) C 1s, (b) F 1s, and (c) O 1s.

XPS analysis was conducted on the uncoated NMC811 and LiF-NMC811 electrodes harvested from half-cells after 100 cycles at 1C to investigate the CEI layer formed on the surface. Figure 9 shows the high-resolution spectra of C 1s, F 1s, and O 1s of the cycled electrodes. The main components of the CEI are byproducts of the electrolyte solvent and salt decomposition, formed during reactions of the electrolyte with the cathode materials at high voltage.^9,10,50^ In the C 1s spectrum (Figure 9a), the intensity from the PVDF binder and carbon black (C–H and C–C) is smaller, while the contributions from C–O and C=O are higher compared to the fresh samples, thereby confirming the accumulation of byproducts on the surface. The C–C peak, albeit lower in intensity, is only distinguishable for LiF-NMC811, which suggests a thicker CEI formation on the uncoated NMC811.^65^ The decomposition of the electrolyte salt (LiPF_6_) and the evolution of the side products on the surface are confirmed by the increased LiF/Li_*x*_PF_*y*_O_*z*_ peak and decrease in the C–F intensity in the F 1s spectrum (Figure 9b).^9,65^ The CEI formation is also confirmed by the substantial C=O/C–O and Li_*x*_PF_*y*_O_*z*_ peaks in the O 1s spectrum (Figure 9c).^10^ The bump at ∼529.8 eV for the uncoated NMC811 electrode is attributed to the formation of the NiO rock-salt phase.^3^ This is in agreement with the CV and EIS results showing the irreversible transition from spinel to rock-salt, which causes higher impedance during longer cycling. Such a peak is not observed for LiF-NMC811. Overall, the post-cycling XPS results confirm the formation and growth of CEI, which impedes charge transfer reactions and results in capacity decline. The implementation of XPS analysis during different cycling periods can give a clearer mechanism as to how the LiF coating inhibits the CEI formation.

**Figure 10:**
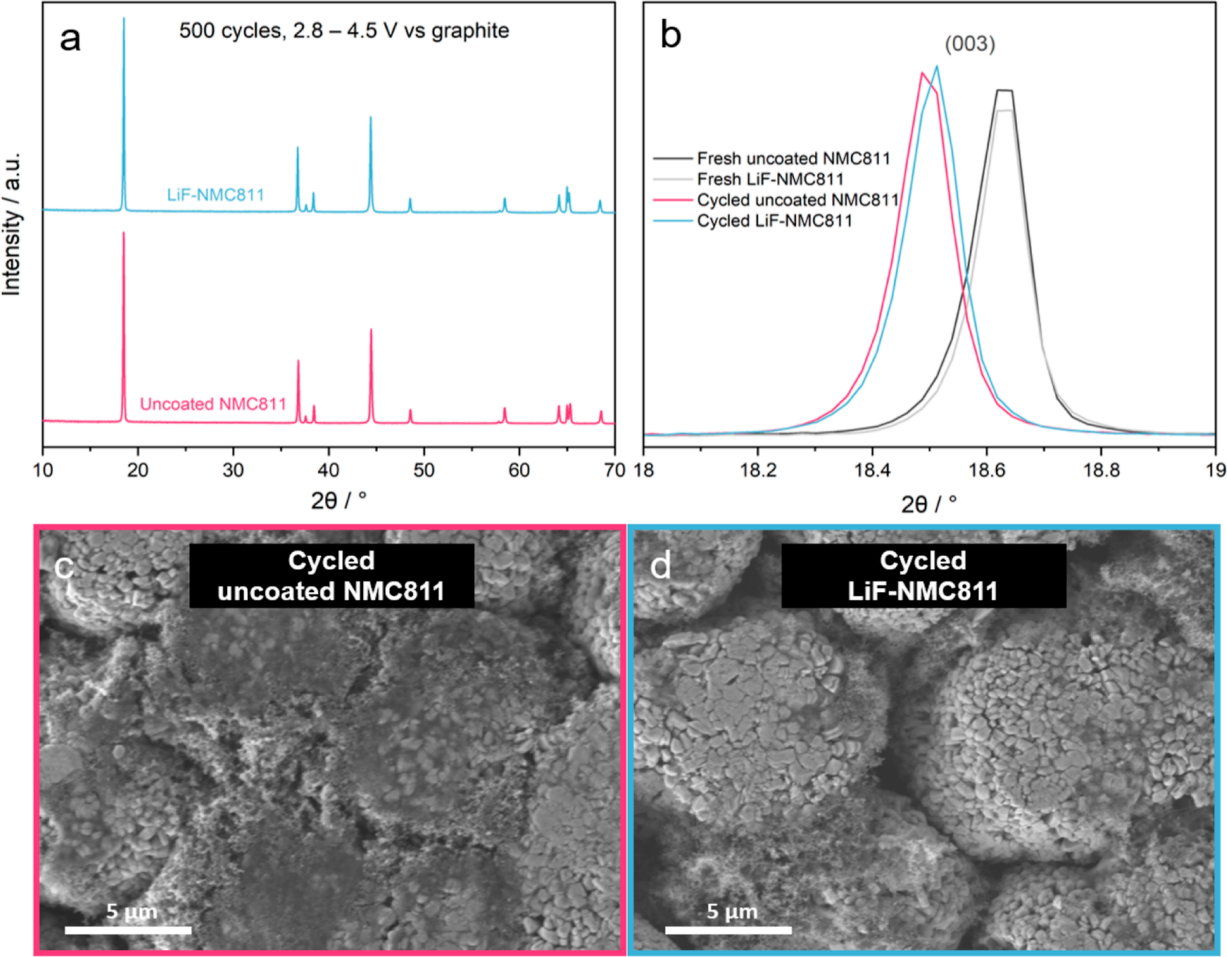
(a) XRD patterns of the uncoated NMC811 and LiF-NMC811 electrodes retrieved from the full-cells after 500 cycles at 1C; (b) magnified view of the (003) peak for the fresh and cycled electrodes; (c) SEM image of the uncoated NMC811; and (d) LiF-NMC811 electrodes retrieved from the full-cells after 500 cycles at 1C.

Figure 10 shows the structural and morphological changes on the cycled uncoated NMC811 and LiF-NMC811 electrodes retrieved from the full-cells after 500 cycles at 1C. XRD patterns (Figure 10a) reveal that the cycled electrodes sustained the layered structure with minimal reduction in peak intensity and no additional phases detected. The samples have a comparable 2θ shift of the (003) peak (Figure 10b), which demonstrates an increase in interlayer spacing after cycling at a high cutoff voltage.^64^ Morphological changes also occur to a lower extent for both samples as there is no significant particle cracking or fragmentation that can be seen in Figure 10c,d. The XRD and SEM results show that there is minimal degradation of the cathode material in the full-cell configuration. A longer cycling duration or higher C-rate may be needed to observe more noticeable changes. Similar results of preserved layered structure and morphology after long-term cycling of an NMC811 cathode material were reported in other studies.^7,54,70,74^

**Figure 11:**
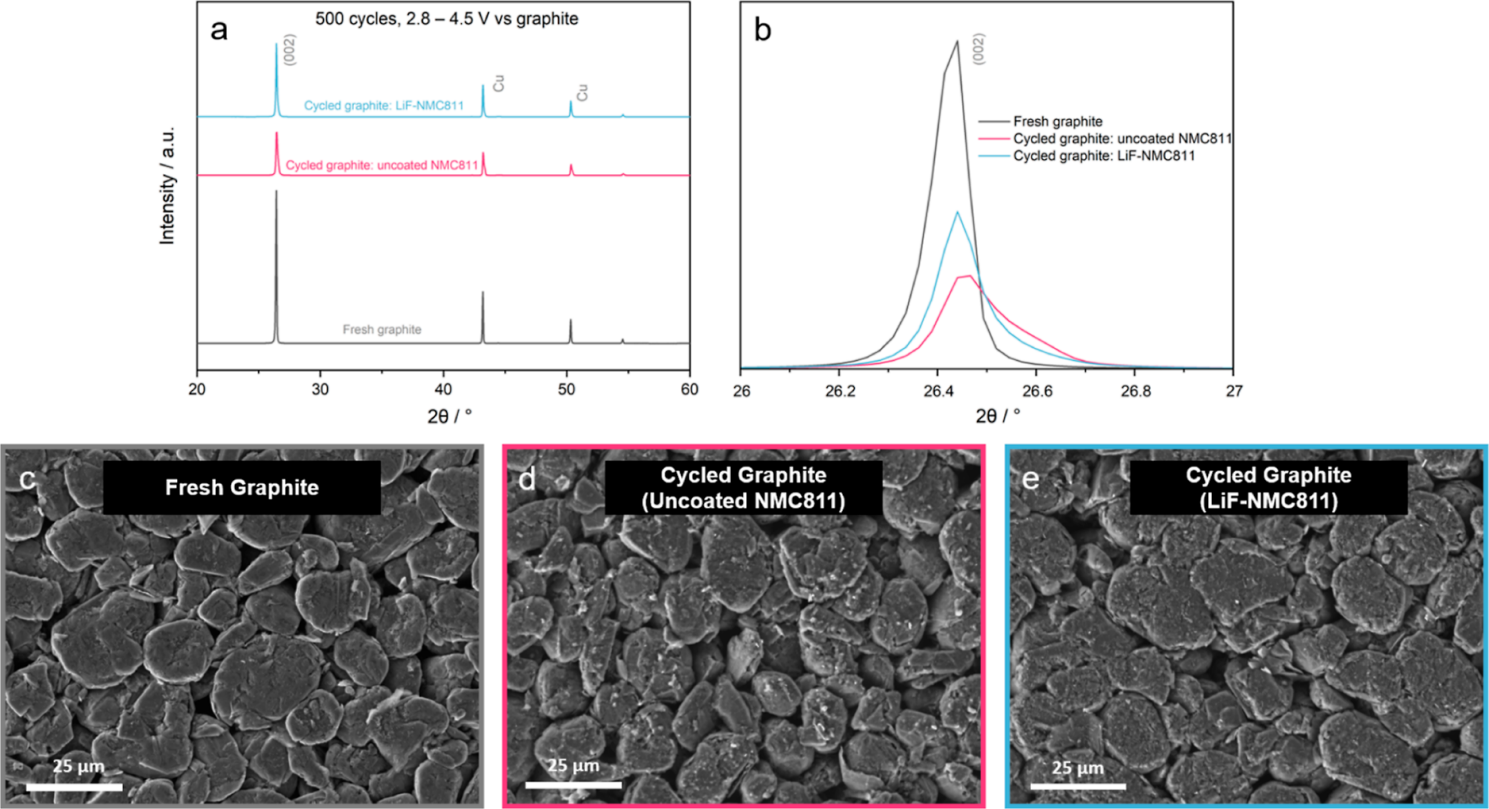
(a) XRD patterns of the fresh and cycled graphite anodes retrieved from the full-cells after 500 cycles at 1C; (b) magnified view of the (002) peak; (c) SEM image of a fresh graphite; and SEM image of graphite retrieved from the full-cell with (d) uncoated NMC811 and (e) LiF-NMC811 as a cathode.

SEI formation is known to be one of the main contributors of capacity fading in full-cells due to the depletion of electrolyte and active Li^+^.^7,74^ XRD and SEM-EDS analyses were carried out on the cycled graphite anodes to probe the development of SEI. Comparing the XRD patterns (Figure 11a), the cycled graphite anodes show reduced peak intensities compared with those of the fresh graphite. A closer look at the (002) peak (Figure 11b) shows a lower intensity for graphite paired with uncoated NMC811. No other peaks can be observed, which may be due to the minimal thickness or amorphous nature of the SEI layer.

Figure 11c,d exhibits how the graphite anode changes from having a smooth fresh surface to a coarse layer after cycling. Transition metal (TM) dissolution of the cathode material and the consequential crossover of the TM ions into the anode side are also reported as drivers of poor battery performance.^2,3,6,7^ The EDS mapping of cycled samples reveals that there is indeed a crossover of Ni from uncoated NMC811 and LiF-NMC811 to the graphite anodes, as shown in Figure S7 (Supporting Information). In addition, more Ni can be found in the graphite cycled with the uncoated NMC811 than in the graphite cycled with LiF-NMC811 as a cathode. This could be due to the stability of LiF against HF, one of the byproducts of electrolyte decomposition, which promotes TM dissolution.^2,12,30,38,52^ These results show that the LiF coating effectively suppressed harmful side reactions between NMC811 and the electrolyte, as well as lessened the TM dissolution in the active material, leading to more reversible electrochemical performance and consequently longer cycle life.

## 4 Conclusions

In this work, LiF coating was successfully developed as an artificial CEI layer on the NMC811 electrode via an atomic layer deposition technique to enforce interfacial stability and improve the cycling performance of high-energy-density LIB. The structural and morphological characteristics of the NMC811 electrode remain unchanged even after the LiF formation. Moreover, the HR-TEM demonstrates that a distinctly uniform nanoscale coating has been deposited on the electrode substrate by ALD. The reported capacity retention values for LiF-NMC811 at high cutoff voltage cycling outperform the uncoated NMC811 and are comparable to studies that utilized a narrower cycling window. LiF-NMC811∥Li is able to deliver a capacity retention of 85% ± 0.6 (161 ± 0.7 mA h g^–1^ at 1C) after 100 cycles, while LiF-NMC811∥graphite retains 88% ± 1.2 (154 ± 1.5 mA h g^–1^) after 500 cycles. The CV and EIS analyses demonstrate that the combination of thicker CEI growth, irreversible transition to rock-salt NiO, and crack formation for uncoated NMC811 contributed to its poor cycling stability in half-cells. However, for the full-cell, SEI formation, transition metal dissolution, and crossover, for the most part, affected the NMC811 stability. The enhanced performance of the LiF-coated electrodes can be ascribed to the suppressed parasitic side reactions between NMC811 and electrolyte, thereby minimizing impedance buildup and active material loss. Of note, despite the added barrier to Li^+^ transport, the rate capability of LiF-NMC811 is comparable to that of the uncoated NMC811, even at high rates. The key findings of this work provide new insights into the development of a highly stable coating as an artificial CEI for a high mass loading and high cutoff voltage Ni-rich NMC cathode material in half-cell and full-cell configurations. As LiF is a major component in CEI, this study also underscores the importance of the CEI role in NMC-based cathodes. Furthermore, the advantages of using ALD in developing coatings directly deposited on an electrode surface with controlled thickness and uniformity have been featured.

## Supporting Information Available

XRD pattern and SEM images of NMC811 active material; elemental mapping of fresh uncoated NMC811 and LiF-NMC811 electrodes; preliminary cycling test of LiF-NMC811 electrodes; equivalent circuit model for EIS analysis; Nyquist plots preceding 1st cycle at 1C; Nyquist plot and fitting results for LiF 250 sample; and EDS spectra of cycled graphite anodes (PDF)
