# Supplementary material for: High Voltage Cycling Stability of LiF-Coated NMC811 Electrode
Source: ACS Appl Mater Interfaces. 2024 Jan 3;16(2):2216–30. doi: 10.1021/acsami.3c14394 (PMC10797589; doi:10.1021/acsami.3c14394)
Supplement: Supplementary file 1 — am3c14394_si_001.pdf [file am3c14394_si_001.pdf]

Supporting Information

High Voltage Cycling Stability of LiF-Coated NMC811 Electrode

Princess Stephanie Llanos[†], Zahra Ahaliabadeh[†], Ville Miikkulainen[†], Jouko Lahtinen[‡], Lide Yao[§], Hua Jiang[§], Timo Kankaanpää[¤] and Tanja M. Kallio[†]*

† Department of Chemistry and Materials Science, School of Chemical Engineering, Aalto University, 02150, Espoo, Finland

‡ Department of Applied Physics, School of Science, Aalto University, 02150, Espoo, Finland

§ OtaNano-Nanomicroscopy Center, Aalto University, 02150, Espoo, Finland

¤ Umicore Finland Oy, 67101, Kokkola, Finland

Email: tanja.kallio@aalto.fi

S1. NMC811 Powder Characterization

The XRD pattern exhibits the appearance of all the peaks associated with $R\bar{3}m$ layered structure without any impurity phases detected. The SEM images show quasi-spherical secondary particles which are 8-10 μm in diameter, composed of densely packed nanosized primary particles.

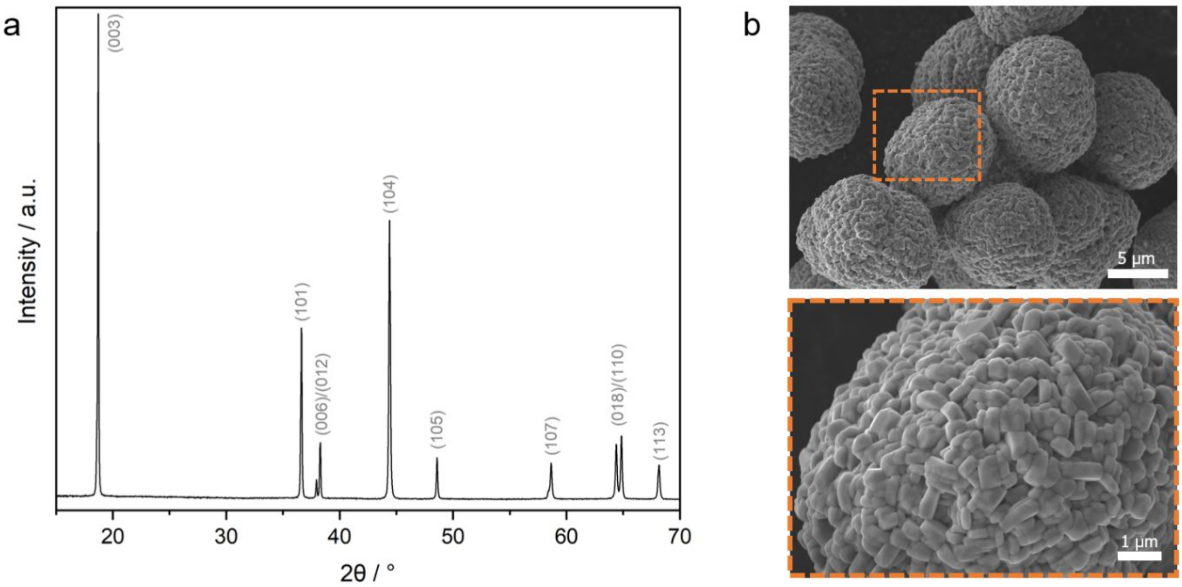

Figure S1. (a) XRD pattern and (b) SEM images of the synthesized NMC811 active material.

S2. Fresh NMC811 Electrode Characterization

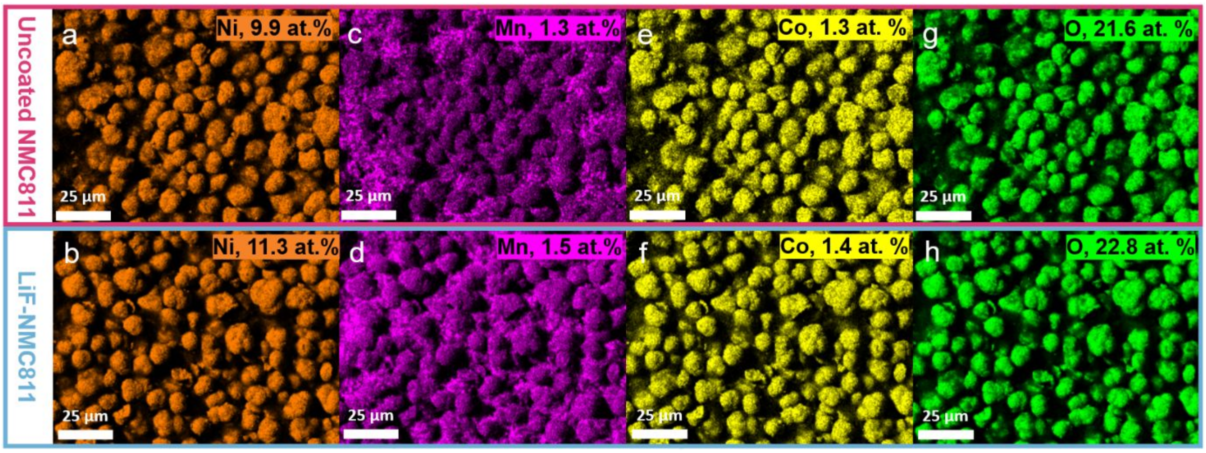

Figure S2. Elemental distribution of (a-b) Ni, (c-d) Mn, (e-f) Co, and (g-h) O in the uncoated NMC811 and LiF-NMC811 electrodes prior to cycling.

S3. Preliminary Cycling Test

150, 200, and 250 ALD cycles were applied on the prepared NMC811 electrode sheets (sample denoted as LiF (ALD cycle number)) to determine an optimal thickness of the LiF coating. LiF 150 delivered the highest specific discharge capacity and capacity retention and has been used for further electrochemical characterization, labeled as the “LiF-NMC811” sample.

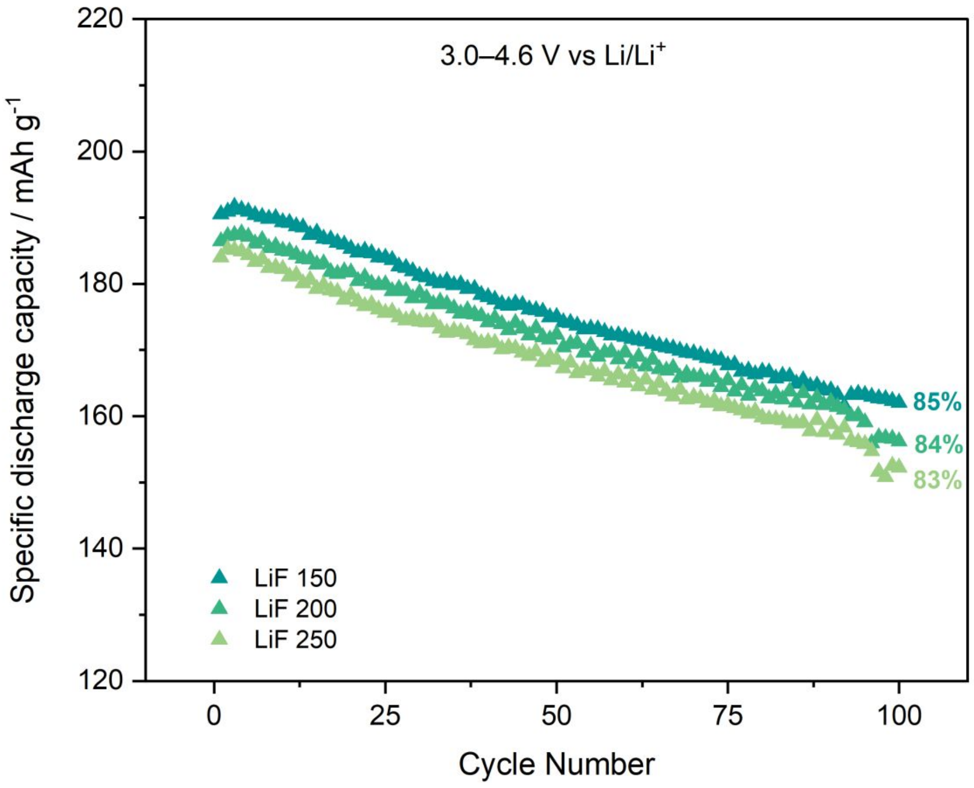

Figure S3. Cycling performance of LiF 150, LiF 200, and LiF 250 tested over 100 cycles at 1C in the potential range 3.0–4.6 V vs Li/Li^+.

S4. Electrochemical Impedance Spectroscopy

The impedance spectra are fitted according to the simplified equivalent circuit shown.

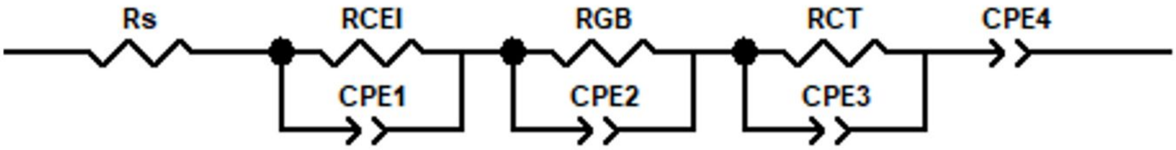

Figure S4. Equivalent circuit for impedance analysis

S5. EIS analysis preceding the 1st cycle at 1C

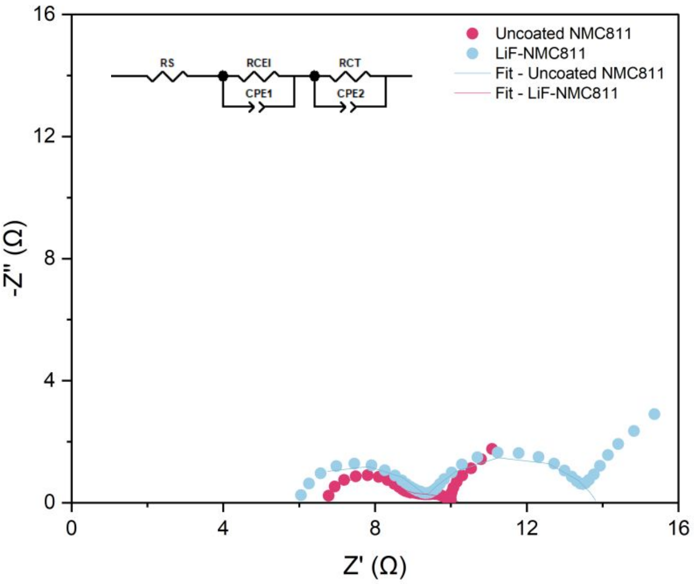

Figure S5. Nyquist plots preceding the 1st cycle at 1C of uncoated NMC811 (pink) and LiF-NMC811 (blue); the inset shows the equivalent circuit for the fitting.

S6. EIS analysis of LiF 250 sample

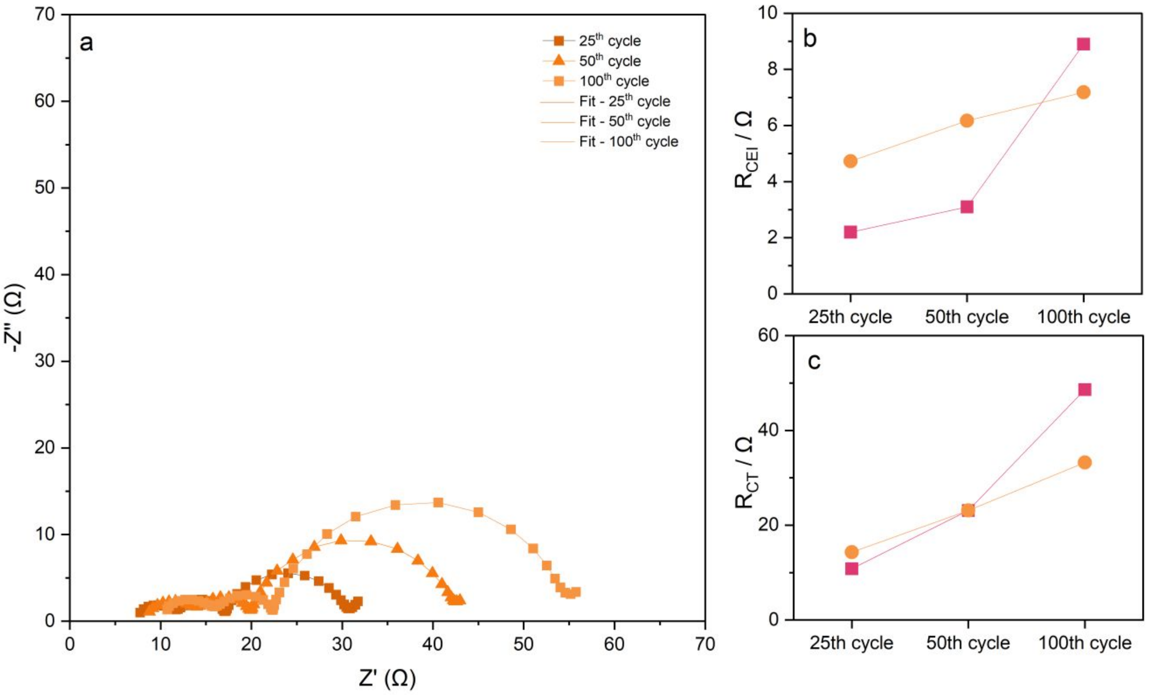

Figure S6. (a) Nyquist plots at 25th, 50th, and 100th cycles of LiF 250 electrode sample; comparison of fitting results (based on Figure S5 equivalent circuit) of impedance spectra for (b) R_{CEI} and (c) R_{CT} of uncoated NMC811 (pink) and LiF 250 (orange).

S7. Elemental Mapping of Cycled Graphite

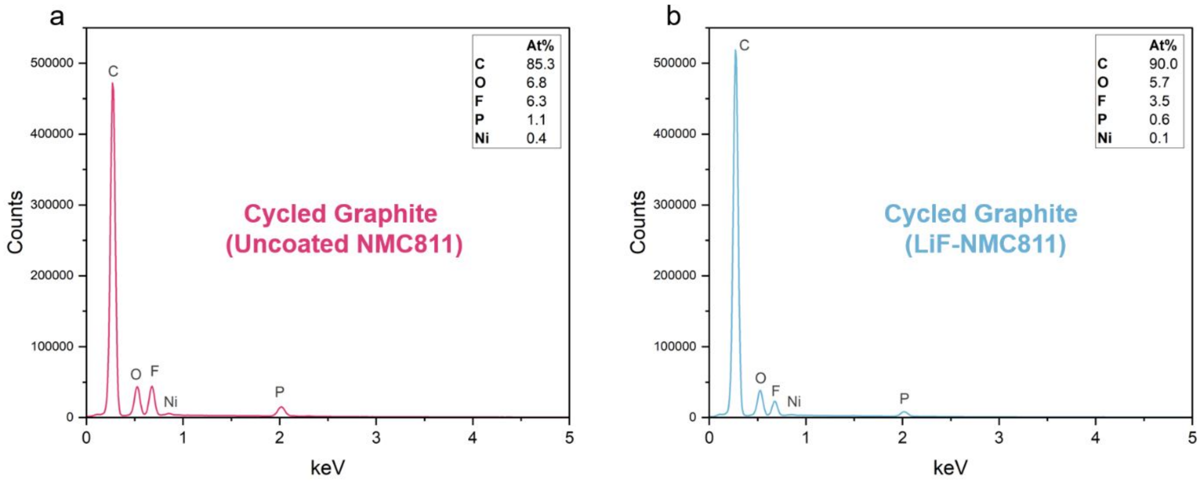

Figure S7. EDS spectra of graphite anodes retrieved from the cycled full cells with (a) uncoated NMC811 and (b) LiF-NMC811 as a cathode.
